# Notes and News

**Published:** 1894-02-17

**Authors:** 


### Notes and News.

Collected and Communicated.

The action of the Director of the Paris *Figaro*, who inaugurated a demonstration of Mons. Hermite's system of electrical sanitation, before the President of the Paris Municipal Council, who is enthusiastically interested in the system, will, it is to be expected, encourage Worthing to seriously consider a trial in its own case. A Bill is before the French Parliament to sanction a huge expenditure on sewage farms for Paris. If Mons. Hermite's system is applicable to a large area, this expenditure will be immensely reduced.

Great excitement has prevailed in medical circles at New York, at the declaration of a young doctor of that city that he had discovered an infallible antidote to opium, morphia, and similar poisons, and moreover that he desired to demonstrate the fact by experimenting on his own person in public. At the meeting formed, amidst the protestations of those present, he drank three grains of morphia in water, immediately followed by a dose of permanganate of potash. No bad results ensued. It is stated that the discovery was accidental. If all tests prove as conclusive as the one so fearlessly offered by the discoverer, Dr. Moore, a most valuable addition to medical science will have been made.

A happy conclusion, as far as the comfort of sick seamen is concerned, has been arrived at in Cardiff. This is the project to abandon the old hulk, the "Hamadryad," which has for years served as a seamen's hospital, and replace it by a building on shore. The rejection of the "Dreadnought" in favour of the present permanent Seamen's Hospital at Greenwich has been so successful that the like arrangement at Cardiff is sure to be popular. The position and surroundings of the old ship have little to recommend them. She is moored in a bleak and exposed spot, surrounded half by marsh and half by foreshore. The vessel is seventy years old, and it may well be held that she has served her time of active service and passive usefulness, and now few will regret the proposed change to an erection on shore.

The International Sanitary Congress was opened on the 7th inst. by M. Casimir Perrier. M. Barrere, French Minister at Munich, was elected President. The Conference decided that its deliberations should be secret, and then adjourned until Tuesday in this week.

H. R. H. the Duke of Cambridge has accepted the presidency of a Committee which has been formed to present a testimonial to Dr. W. H. Dickinson, on his retirement from the office of Senior-Physician to St. George's Hospital, of which His Royal Highness is a vice-president. Among the members of the committee are the Duke of Westminster, the Earl of Cork and Orrery, Mr. Shaw Stewart and Colonel Haygarth, vice-presidents of the Hospital, Mr. J. R. Mosse, treasurer, Sir Henry Acland, Admirals Sir George Willes and Sir W. Houston Stewart, Sir George Humphry, Sir Francis Laking, Surgeon-General Cornish, and a number of Dr. Dickensons's past and present colleagues, and pupils and former students of the St. George's Medical School.

We have regarded the question of a joint isolation hospital for Richmond, Heston, and Isleworth as a settled matter, but it appears that the union is not yet a *fait accompli*, as Twickenham has issued a protest. Heston and Isleworth must in any case have their hospital, but Twickenham objects to Richmond being taken in, as the route for Richmond patients would run through part of Twickenham. The Local Board of this place have called the attention of the Middlesex County Council to the matter. The chairman of the Council at the last meeting showed very strongly his feeling of opposition to the scheme, which, however, was unusually firmly upheld by others present, especially by representatives of Isleworth and Heston, who announced that they could not be stopped sharing their hospital with Richmond if they pleased to do so. The meeting resulted in a resolution to inquire and report on the matter.

The results of the Opium Commission, now sitting at Indore and at Ahmedabad, cannot fail to be looked for with extreme interest by the public. Such partisanship has been awakened by the opium question, and such bitter word battles have been fought over it, that all must look to the Commission for ammunition for the one side or the other. In England we point to sensational accounts of opium dens, and the large revenue derived from a pernicious commodity by the Government. In India the tendency is to say "Look first at home—opium is harmless in comparison to the consumption of spirits, which legislature cannot check." In malarial districts opium is regarded as almost essential by a large number of the medical profession, and the whole crusade against opium is regarded by the majority of those who have studied the question as a well-meaning but sensational absurity. The first witness before the Commission, the Governor General's Agent for Central India, stated that he considered the prohibition as likely to produce civil war, and Colonel Robertson, Political Agent at Baghelkand, whilst holding the evil as much exaggerated, expressed the same views of the political consequences. At the second meeting, interference was objected to from various points of view, and the witnesses were all in favour of leaving the matter untouched.

